# Designing a knowledge translation tool for women’s health research in the U.S. Military Health System

**DOI:** 10.1186/s12961-023-01002-9

**Published:** 2023-06-08

**Authors:** Miranda Lynn Janvrin, Jessica Korona-Bailey, Tracey Perez Koehlmoos

**Affiliations:** 1 Uniformed Services University of the Health Sciences, 4301 Jones Bridge Road, MD 20814 Bethesda, United States of America; 2 The Henry M. Jackson Foundation for the Advancement of Military Medicine, 6720 Rockledge Dr., MD 20817 Bethesda, United States of America

**Keywords:** Military health services, Women’s health, Key informant interviews, Knowledge translation, Unintended pregnancy, Unplanned pregnancy

## Abstract

**Background:**

Current United States Department of Defense (DoD) estimates indicate that women comprise 17% of the total active duty component. Despite this, the specific health needs of service women have often been neglected. The Center for Health Services Research (CHSR) at the Uniformed Services University (USU) has been working to create a portfolio of rapid research synthesis briefs on topics including, but not limited to reproductive health, infertility, pregnancy loss, and contraceptive use among active duty service women. The goal of these briefs is to condense and translate the existing research literature for a non-academic audience. The aim of this study is to evaluate the utility of the research briefs to inform decision making around service women’s health issues and impart an overall understanding of the current literature surrounding these topics to a non-academic audience.

**Methods:**

Adopting a previously tested knowledge translation evaluation tool, we conducted a series of key informant interviews in July–August 2022 with decision makers in the Military Health System and the US DoD to elicit feedback regarding the overall utility of the research brief, as well as its ability to meet standards of usefulness, usability, desirability, credibility, and value.

**Results:**

We interviewed a total of 17 participants of a diverse range of healthcare occupations and educational backgrounds, but all currently were working within the Department of Defense in support of the Military Health System. User feedback on the research brief was thematically evaluated based on the predetermined themes of usefulness, desirability, credibility, value, and two emergent themes—findability and language.

**Conclusions:**

This study allowed us to gather key insights from decision makers to better tailor future iterations of our research brief toward rapidly disseminating information for improving the healthcare and policy of active duty service women. The key themes ascertained from this study may help others when adapting their own knowledge translation tools.

## Background

Current United States Department of Defense (DoD) estimates indicate that there are over 369,000 active duty service women comprising 17% of the total active duty component [1, 2]. Despite this, the specific health needs of service women, especially reproductive care, have often been neglected [2]. Treatment options for reproductive conditions are limited by location, accessibility, cost, and even rank [2, 3]. This is especially true for women deployed to remote areas or aboard ships, for whom access to reproductive care is highly limited [1–3].

The Center for Health Services Research (CHSR) at the Uniformed Services University (USU) has been working to create a portfolio of rapid research synthesis briefs, on topics including, but not limited to reproductive health, infertility, pregnancy loss, and contraceptive use among active duty service women. The goal of these briefs is to condense and translate the existing research literature for a non-academic audience. This process is known as knowledge translation, defined as a, “dynamic and iterative process that includes the synthesis, dissemination, exchange and ethically sound application of knowledge to improve health, provide more effective health services and products, and strengthen the health care system” [2]. Knowledge translation is meant to assist in informing decision makers about evidence based practices and policies, as well as to impart an understanding of the current research for targeted issues [4–6].

To facilitate this process, the CHSR team has developed a knowledge translation tool based on review of others in use by similar organizations. The tool is a two page research brief tailored to the subject matter. Research has shown that on average there is a 17-year difference between the generation of new knowledge and the practical implementation of that knowledge, highly detrimental for military, veteran, and civilian health care organizations [7, 8]. Knowledge translation tools are designed to facilitate the knowledge translation process, improving the uptake of knowledge into practice [4–6, 9]. However, the current tool has not yet been optimized to our target population and thus it is difficult to ascertain its utility. The aim of this study is to evaluate the utility of our knowledge translation tool to inform decision making around service women’s health issues, and impart an overall understanding of the current literature surrounding these topics to a non-academic audience.

## Methods

The CHSR team adapted the user test package developed by Cochrane Norway to assess the utility of our rapid synthesis research briefs and proposed a series of key informant interviews with decision makers in the Military Health System (MHS) and the DoD to elicit “think-aloud” feedback regarding the overall utility of the research brief, as well as its ability to meet standards of usefulness, usability, desirability, credibility, and value [10–13]. The aim of these interviews was to obtain a better understanding of the user experience, observe any problems that the user encounters while using the tool, and inform future changes in design and content on the basis of the structured observations and participant feedback. Two researchers conducted interviews with members of the Women and Infant Clinical Community, the Services (Army, Navy, and Air Force), and representatives from TRICARE. Interviews were conducted from July 15, 2022–August 5, 2022. During the interview, the researchers utilized a think-aloud semi-structured interview guide. One researcher served as the interview guide while the other took notes.

The research brief used for evaluation was a two-page synthesis of the literature around miscarriage in active duty service women. It consisted of seven parts: a title page with logos and a disclaimer, a “bottom line up front (BLUF)” section that provided a two sentence summary of the synthesis, a “question” section that defined the question to be answered, a “why the issue is important” section, a methods section, a findings section, and a table that summarized each of the studies included in the synthesis.

Participants were asked background questions and initial impressions. The interviewer then guided them through each part of the document, prompting them to think aloud. The interview guide was based on the SUPPORT User Testing framework developed by Cochrane Norway for user experience with six facets: credibility, understandability, usability, usefulness, desirability, value [10–12]. The participant was then asked for feedback and any additional comments. Notes were collated and de-identified to ensure confidentiality. The researchers read through the notes and conducted deductive thematic analysis of responses based on the six facets. The researchers also identified barriers and facilitators to understanding and sorted findings according to key themes. This study was found exempt by the Institutional Review Board of the Uniformed Services University of the Health Sciences.

## Results

We interviewed a total of 17 participants of a diverse range of healthcare occupations and educational backgrounds but all currently were working within the DoD in support of the MHS. The majority of participants’ highest education completed was a doctoral degree, the remaining participants’ highest education completed was a master’s or bachelor’s degree. Participant occupations were comprised of department chiefs, directors, deputy department chiefs or directors, nurses, nurse consultants, physicians, program directors and managers, and researchers.

When asked where participants seek information when making policy decisions, the most commonly given answers were academic journals, websites, or colleagues.

### Thematic review

Identified themes include the six user facets identified by Cochrane Norway: credibility, understandability, usability, usefulness, desirability, value [10–12]. Two emergent themes were discovered during the thematic review: language and findability.

### Credibility

The majority of participants responded that they would trust the information given in the brief, without having read it critically, while a few said they would hesitate to trust it. Of those that said they would trust it, reasons given were the logos on the front page and the references section.

“…the chart references sources and the references at the end of the file. That to me lends credibility because when you look at this, this is not just the author’s opinion.”

A couple participants attributed the credibility to the tables in the findings section. A few admitted that they were willing to trust the information based on the authors listed on the title page, saying:

“One, there is helpful information on references to show what was leveraged and two, knowing the source of the information. USUHS is very credible I know the quality of the work. Seeing the references and citations makes it even more credible.”

Some of the participants who were hesitant to trust the information in the report said it was due to the lack of a quality assessment score for the included studies. A couple said it was due to the age of the citations included. Others said it was due to the report appearing to lack depth on account of its short length. One concluded that it was because they take nothing at face value.

### Usability

Participants were guided through each aspect of the research brief and asked for their impressions, feedback, and anything that stands out as a barrier or facilitator to understanding. Facilitators to understanding included the use of colored boxes to organize the information contained in the research brief, with one participant saying “…boxes on the second page that stand out are great.” Another facilitator included the addition of a BLUF section that gives a very brief overview of the key findings, with participants saying, “If I need something quick, I can go to it, I can pick the BLUF,” “BLUF is nice quick and dirty on it,” and that a “BLUF is always welcome.” Another commonly cited facilitator was the chart containing relevant and recent references, with participants saying, “Nice to have references listed with study design and key findings,” and “Like the presentation, love tables.” The final facilitator identified was the overall short length of the research brief with one participant saying, “…sometimes research results can be lengthy—I likes that this is not lengthy.”

Barriers to understanding were also assessed and included the lack of a direct connection between the topic of the research brief and its impact on military readiness with participants asking, “…why is this important to the military?” and commenting that “Since talking about active duty service women, there is no connection back to how it impacts the mission of the unit.” Another key barrier to understanding included the lack of a literature quality indicator, with one participant directly saying, “a quality analysis would be helpful.” A further barrier to understanding was the formatting of the methods section with one participant commenting that “…it looks like it should include the findings the way it is laid out-looks like it should be a summary box.” The final barrier identified was the lack of a glossary of terms used throughout the brief, with one participant saying, “…there is a lot of vague use of words without definitions.”

### Understandability

All participants said that the report was “generally easy” to understand. Reasons given for this included the overall formatting and brevity of the brief, with one participant saying, “Short, good design, this contributes to readability,” and another saying, "The formatting and the boxes and tables really helped [as well as the] bullet points.” Another cited that the brief “Steered clear of jargon,” which aided understanding. Another responded that the use of the table to display the findings facilitated understanding, saying, “I think using a chart is helpful and inclusion of references so people can know where to find information if they want more.”

### Usefulness

When asked if this brief would be useful to them if they needed to make a policy decision on this topic, the majority of participants said it would, a few said it would not, and a few others said it might. For those who said it might, the most given reason was that it could be a source of information, but not the sole source, saying, “I think it would be one piece of information. We couldn’t solely make a decision based on this,” and another saying, “[The research brief] could be part of making a decision but lacks breadth to be sole source.” Another participant said that it would be dependent on the policy decision being considered, highlighting that it would not be as useful for more specific questions.

“I think relative to how beneficial; it would depend on the policy being considered. If the policy was broad … it would be helpful. If it was specific decision, the usefulness would depend on the policy decision being made.”

Those who said that it would not be useful said that there was not enough evidence or scientific rigor to be useful.

### Desirability

Participants were asked if, to the extent that you could like a brief, they liked it, most said that they did like the brief, and an additional participant said that they “mostly liked it”, and another that it was simply “okay.” When asked to elaborate, the most common reasons participants gave were that they liked that the report was concise, the formatting of the report was easy to read, and the references within the table were helpful. Those who said that they did not like the report felt that it “did not have enough evidence to back up the claims made” and “lacked scientific rigor.”

When asked for suggested changes to the brief to increase its desirability, the most common responses were to add more depth and specificity, to add recommendations for future research and implications for policy making, and to condense the content down to one page.

### Value

To determine if the brief was valuable to participants, they were asked if a series of briefs of this type would be helpful for a person in a position like theirs. Nearly all participants reported that they would be, while a couple said maybe. Some participants said that there is always a need for a succinct review of current topics in the research literature. One participant said that it would be especially beneficial to those in leadership positions, as lengthier reviews are too time consuming. Of those who said maybe, they highlighted the need to show a direct translation to military readiness.

The participants were then asked how the brief could be made more valuable to them. Suggestions overlapped with barriers to understanding from the usability theme and included adding a quality appraisal, adding a glossary of key terms, and to add recommendations for future research.

### Language

The first emergent theme that came out of our thematic review was that of language. Our participants found that while overall the research brief steered clear of jargon, there were certain terms that hampered their understanding. Specifically, these terms were “rapid synthesis” cited by several participants, “grey literature” cited by a few participants, and the acronym “BLUF” cited by a few other participants.

### Findability

The second emergent theme was findability. When asked where participants would expect to find a research brief like this one, or a series of research briefs like this one, all participants reported that they were unsure. One participant said, “That is difficult, one of the biggest problems, we don’t have anywhere to look at reports like these especially for a [Department of Defense] generated topic.” Common answers included colleagues or the CHSR.

## Discussion

We adapted a previously demonstrated evaluation tool to assess the utility of our rapid research synthesis briefs on service women’s health in the MHS [10, 11]. Knowledge translation tools like our research briefs exist to aid in the process of disseminating research results to decision makers [4–6], and the easiest way to determine how decision makers would prefer to receive this kind of information is by involving them in the iterative creation process. Inclusion of members of our target population allowed us to ensure that any takeaways gained from this process would allow us to tailor our research briefs to the decision makers we are trying to reach. The greatest takeaway from our interviews with participants was the level of interest from decision makers within the DoD for knowledge translation materials of this kind. All of our participants, from a diverse range of positions, expressed interest in research briefs of this kind during the interviews. Our emergent theme of findability also highlighted that there is a gap in dissemination of knowledge translation materials of this kind within the DoD.

Some of the facilitators of understanding, value, and desirability were things that we had expected and were included in the design. Previous literature on knowledge translation tools highlighted that brevity is key in facilitating understanding. With this in mind, we aimed to keep the research brief short in length, which was appreciated by our participants, especially decision makers who admitted themselves that they do not have time to read lengthy research articles, confirming this finding [10–12]. Refraining from using technical jargon was also another intentional choice that was confirmed by our participants to aid in their understanding and the usefulness of the research brief overall. Despite efforts to avoid jargon, language still emerged as a common theme, with three terms in particular being cited: “rapid synthesis”, “grey literature”, and “BLUF.” Further iterations of the research brief will seek to remove or define these terms. While the term “BLUF” was questioned, the section itself providing a short summary of the key findings was considered a welcome inclusion by most participants.

The consensus was that, especially for decision makers, having a one or two sentence summation of findings allows them to determine if they need to read further and possibly disseminate the findings from the brief themselves. Further iterations will include the BLUF box, but determine if there is a better term to denote this section (e.g. Summary or Overview). Another facilitator of understanding cited by participants was the inclusion of a table that summarized the literature, allowing them to access each article themselves as needed. While further iterations will seek to include the table describing the literature, it is unclear if this approach will be possible for topics in the literature that are more well established. Formatting choices appeared to be a double edged sword, with the colored boxes being appreciated by participants, but the choice of content within the boxes being questioned. Further iterations will carefully consider organization and formatting based on this feedback.

Barriers to understanding, value, and desirability will also be considered carefully for further iterations of the research brief. Connecting the topic to military readiness was key to several participants who felt that without that connection it would be difficult to make a decision based on the research brief for a military population and its inclusion is necessary to ensure usefulness and value. The addition of a literature quality grade was also seen as necessary to provide value, as some participants felt they would be unable to make a decision from conclusions based on literature without knowing it was high quality research. Another key to desirability in particular was the need for a recommendations and future research section, with participants feeling that they had been told about a topic, but unsure what steps the authors suggest be taken next to address the topic.

The primary limitation of our study is that it is unclear if knowing that the interviewers were involved in the creation of the research brief had an impact on participant responses. However, withholding this information may have impacted participant’s view of the credibility of the research brief, which is why we chose not to in this case. Another limitation is that we only included 17 participants, though we feel that this number was robust enough to reach saturation and produce meaningful findings. However, a strength of our study was the ability to adapt and successfully employ an interview guide was based on the SUPPORT User Testing framework developed by Cochrane Norway [11].

Further research should look into the use of knowledge translation tools for other topics within women’s health and the development of best practices for knowledge translation within the DoD.

## Conclusions

As the goal of knowledge translation is to aid decision makers in making informed policy, it is mutually beneficial to consult with the target demographic to understand how they prefer to receive information. This study allowed us to gather key insights from decision makers to better tailor future iterations of our research brief toward rapidly disseminating information for improving the healthcare and policy of active duty service women. The key themes ascertained from this study may help others when adapting their own knowledge translation tools.

## Data Availability

All information pertaining to our research is contained within the manuscript; no external datasets were used.

## Abbreviations

BLUF: Bottom line up front
DoD: United States Department of Defense
CHSR: The Center for Health Services Research at the Uniformed Services University for the Health Sciences
MHS: The United States Military Health System
USU: The Uniformed Services University for the Health Sciences
